# Outpatient Antibiotic Prescriptions in Pregnant Women in Małopolska Province

**DOI:** 10.3390/antibiotics10010014

**Published:** 2020-12-26

**Authors:** Anna Różańska, Agnieszka Pac, Estera Jachowicz, Dorota Jaślan, Małgorzata Siewierska, Jadwiga Wójkowska-Mach

**Affiliations:** 1 Microbiology, Jagiellonian University Medical College, 31-121 Kraków, Poland; estera.jachowicz@doctoral.uj.edu.pl (E.J.); dorota.jaslan@gmail.com (D.J.); mbmach@cyf-kr.edu.pl (J.W.-M.); 2 Department of Epidemiology and Preventive Medicine, Jagiellonian University Medical College, 31-121 Kraków, Poland; agnieszka.pac@uj.edu.pl; 3 Department of Ophthalmology, St. Rose Hospital, 30-394 Kraków, Poland; tsiewi@wp.pl

**Keywords:** pregnancy, antibiotic therapy, defined daily dose, infection prevention

## Abstract

Background: excessive and incorrect use of antibiotics is a common event that contributes to increased bacterial resistance to antibiotics. Based on the European Centre for Disease Control and Prevention (ECDC) European Surveillance of Antimicrobial Consumption (ESAC)-Net reports, outpatient antibiotics prescribing in Poland, is higher than the European average rate. However, ECDC reports do not provide data on consumption in specific patient groups. Additional studies focused on selected patient groups are needed for a more detailed analysis. The use of antibiotics during pregnancy is inevitable due to the proven risk of complications in this period, perinatal infections, and infections in newborns, in the situation of frequent infections of the genital tract, urinary tract, and asymptomatic bacteriuria in pregnant women. The aim of this study was to analyze the use of antibiotics/the implementation of antibiotic prescriptions ordered by gynecologists during pregnancy in pregnant women in Małopolska Voivodeship, with particular emphasis on demographic data and drug groups. Material and methods: the analysis used data on the reimbursement of antibiotics from the Małopolska Health Fund for 2013–2014. The database contained information of a demographic nature: age, place of residence, date of birth, as well as information on the type of drug, and the number of packages purchased by the patients. Results: the study included 67,917 women. During pregnancy, 23.6% filled their prescriptions for antibiotics. In the first trimester, the percentage of women filling their prescriptions for antibiotics was the lowest and amounted to 7.8%, in the second and third trimesters, these were 9.0% and 11.2%, respectively. The inhabitants of rural areas bought antibiotics more often (23.5%). Most frequently, antibiotics were purchased by women under 18 years of age (32.1%), but the use of antibiotics in women aged 18–34 and over 35 was similar, amounting to 23.8% and 22.7%, respectively. Most often (94.78% of cases) monotherapy was used, including beta-lactams and beta-lactams with an inhibitor (67.3%), then second-generation cephalosporins (13.5%), macrolides (14.73%), and others. Prescription of two different preparations was found in 5.06% of cases, three (0.19%), and four or more (0.03%). Conclusions: due to the fact that studies on antibiotic therapy in pregnant women in Poland are rare, it is impossible to assess the situation thoroughly. Additionally, in Poland, the prevalence of gestational infections is unknown, and their diagnostics is important, especially in relation to microbiological diagnostics and targeted treatment. However, the present results indicate the need to implement broad health promotion programs, accessible to all women, also those living outside large cities, particularly promoting the prevention of urinary tract infections and reproductive health in general.

## 1. Background

Excessive and incorrect use of antibiotics is a common event that contributes to the growth of bacterial resistance to antibiotics [1]. The results of the European Surveillance of Antimicrobial Consumption (ESAC) project implemented in European countries by the Healthcare-associated Infections Surveillance Network (HAI-Net), European Centre for Disease Prevention and Control (ECDC), indicate a problem with the use of antibiotics in Poland in the open health sector. The average consumption in Poland in 2009–2018 was high: it ranged from 18 to 23.8 DDD/1000 people/day, and showed an upward trend, while the corresponding values of the European average were 10 to 40% lower, with a slight (0.3%) downward trend [2]. However, the antibiotic consumption in specific patient groups is not reported in this project. Obtaining such information requires prospective or retrospective, targeted studies, which are not very common. In the ECDC study, analyses were carried out not only with regard to the consumption of all antibacterial drugs, but also taking into account the consumption of individual groups of antibiotics. A closer analysis shows that the use of specific groups of antibiotics or their use in specific populations may be problematic. According to ECDC reports, the consumption rate of trimethoprim with sulfamethoxazole was particularly high (2012)—twice as high as the European average [3], and in 2018, twice as high consumption was recorded in the group of "macrolides, lincosamides, and streptogramins" and in the group "other" (classification used in ECDC reports) [2]. In the case of trimethoprim/sulfamethoxazole, an interesting observation was noted in the study by Pomorska-Wesołowksa et al., indicating extremely high resistance of *Staphylococcus aureus* to this antibiotic in patients over 80 years of age [4]. The analysis of trends and the structure of antibiotic consumption reported in the ESAC program coordinated by ECDC also showed that the consumption of fluoroquinolones in Poland is higher than the European average values and with an upward trend [5]. These data confirmed the necessity of analysis of antibiotic consumption in specific patient populations. Data on antibiotic consumption may also be used, especially for a given patient population, to identify areas of care that require intervention or modification of existing infection prevention procedures in specific patient populations. For example, the analysis of data on antibiotic prescriptions for women, in the period of 30 days after childbirth, confirmed, with a very high probability, the thesis about the lack of effective post-discharge surveillance after cesarean sections [6,7]. Additionally, the analysis of the use of antibiotics within 30 days of childbirth showed a significantly higher use of antibiotics in the youngest women and rural residents, which indicates which groups of patients require special attention. In a multicenter study in southern Poland, fewer than one patient per 100 had surgical site infection (SSI) after cesarean delivery, while European data indicate 1.8% [8]. 

Antibiotics are among the medications most commonly prescribed for pregnant women [9]. In the United States, pregnant women make up about 10 million people, i.e., about 3% of the population [10]; in Poland, in recent years, the number of deliveries has been around 400,000 per year (1% of the population) [11]. The use of antibiotics in this group of patients is inevitable due to the proven risk of complications in the course of pregnancy, perinatal infections and infections in newborns, in the situation of frequent infections of the genital tract in pregnant women [12,13,14,15], and urinary tract and asymptomatic bacteriuria [16]. Infection surveillance, including effective treatment, is also associated with the control of perinatal infections, for example, bacterial vaginosis is one of the most important prognostic factors for chorioamnionitis [17]; furthermore, chorioamnionitis is a significant risk factor for morbidity and mortality in neonates [18]. Antibiotics, therefore, play an important role in improving and promoting the health of pregnant women. Nevertheless, as with other therapies, overuse can be counterproductive.

The most important rules concerning antibiotic usage during pregnancy comprise usage of medications only if absolutely indicated, avoiding, if possible, initiating therapy during the first trimester, selecting a safe medication (which is often an older drug with an approved track record in pregnancy), preferring, wherever possible, single-agent therapy to combination therapy. Moreover, narrow-spectrum antibiotics are preferred over those with a broad spectrum for the treatment of established infection and intrapartum GBS chemoprophylaxis. These rules also include the use of the lowest effective dose and discouraging the use of over-the-counter drugs, which may interfere with the efficacy and/or metabolism of prescription medications [19]. 

The aim of this study, which is a continuation of the cited study, was to analyze the use of antibiotics/filling antibiotic prescriptions ordered by gynecologists during pregnancy, in pregnant women in Małopolska Voivodeship, with particular emphasis on demographic data and drug groups.

## 2. Materials and Method

The analysis used retrospectively collected data on the reimbursement of antibiotics from the Małopolska National Health Fund (NHF) for 2013–2014 in relation to data concerning deliveries reimbursed by this payer. For patients who gave birth in 2013 and 2014, owing to the unique system identification number, data on filling the antibiotic prescriptions ordered by gynecologists in the course of the nine months preceding childbirth were checked. The database, anonymized by the National Health Fund, contained selected information of a demographic nature: age and place of residence, date of delivery (allowing analysis by trimester), as well as information on the type of drug and the number of packages purchased by the patients. The NHF reimbursement system is based on the Diagnosis-Related Groups (DRG) and for different types of deliveries following codes are gathered: N01, N02, N03, N09, N11, N13. N01 represents delivery, N02– multiple or preterm births, N03– pregnancy or fetal pathology, with childbirth > 5 days, N09–severe pathology of pregnancy with childbirth, including extended diagnosis, comprehensive treatment > 6 days, N11– severe pathology of pregnancy with childbirth, including extended diagnosis, comprehensive treatment > 10 days, N13–severe pathology of pregnancy ended with operative delivery > 3 days. Therefore, the database does not allowed to distinguish multiple and preterm birth, and does not include, and the analysis does not encompass, miscarriages. For analysis, three types of birth were taken into account: single child (N01), multiple or preterm birth (N02) and pregnancy pathology (N03, N09, N11, N13 together).

Antibiotic consumption was expressed as the percentage of women with prescription and the aggregate sum of defined daily doses (DDD) according to the ATC/DDD (Anatomical Therapeutic Chemical) system of the World Health Organization, group “J01” [20]. Only antibiotics for systemic use were taken into account—no antifungal (J02), antimycobacterial (J04), or antiviral (J05) drugs were included in the analyses. The data referring to the quantities of antibiotics used are expressed in the World Health Organization (WHO)-recommended defined daily doses (DDDs, access valid on 27 November, 2020: http://www.whocc.no/atc_ddd_index/). 

The one-time implementation (due to one case of infection) was the purchase of antibiotics based on prescriptions with different purchase dates, if the interval between them was not longer than 14 days. With the date of delivery known, cases where prescriptions were filled by individual women up to 280 days before delivery were taken into account, including the first trimester—up to 182 days, the second trimester, from 92 to 182 days, and the third trimester—90 days or less before delivery. The results are presented in the form of the number and percentage of women taking antibiotics during pregnancy in each trimester and calculated mean values with standard deviation for DDD per one woman taking antibiotics and per 100 pregnant women. The frequency of taking antibiotics depending on the selected characteristics of the subjects and childbirth was compared using the Chi2 test, while the distribution of the number of doses using the nonparametric Kruskal–Wallis test due to the lack of normal distribution of these variables. The differences for which *p* < 0.05 were considered statistically significant, but in the case of multiple comparisons, the Bonferroni correction was applied. The analysis used the IBM SPSS ver. 26.

This work was approved by the Bioethics Committee of Jagiellonian University (approval no. 122.6120.29.2017). The study was based on the data gathered during routine patient care and the analysis did not include any individual participant’s data. As a result, no consent statements were required from participants. The study in this form was approved by the local Bioethics Committee of Jagiellonian University.

## 3. Results

The study included 67,917 women, 23.6% of whom filled their prescriptions for antibiotics during pregnancy, the mean DDD per patient was 44.7 (Table 1). In the first trimester, the percentage of women fulfilling their prescriptions for antibiotics was the lowest and amounted to 7.8%, in the second and third trimesters, it was slightly higher: 9.0% and 11.2%, respectively. Some women filled prescriptions in more than one period, hence the data from trimesters do not add up to the overall percentage. Antibiotics were purchased significantly more often by rural women, compared to city dwellers, i.e., 23.5% and 21.9% of city dwellers, in cities up to and above 100,000 of residents, respectively, redeemed the prescription for antibiotics vs. 24.4% of female rural residents (*p* < 0.001) (Table 2). A similar observation, i.e., the most frequent filling of prescriptions by rural residents and the youngest women, also applies to individual trimesters (Table 3, Table 4 and Table 5).

Most often, antibiotics were purchased by women who delivered in tertiary referral hospitals and with a multiple or preterm birth. In the case of hospital preferentiality, these differences were statistically significant in each case, and in women with multiple or preterm childbirth, the lack of statistical significance was found only in the third trimester. Taking into account the entire period of pregnancy, the average number of purchase episodes/probable number of infections in one woman was 1.3 (SD 0.70), and in individual trimesters, it was 1.1 (SD from 0.24 in the first trimester to 0.38 in the second and third trimesters). 

Most often (94.78% of cases) monotherapy was used, including beta-lactams and beta-lactams with an inhibitor (67.3%), followed by second-generation cephalosporins (13.5%), macrolides (14.73%), and others. Prescription of two different preparations was found in 5.06% of cases, three (0.19%), and four or more (0.03%).

## 4. Discussion

In the studied group of women giving birth in Małopolska Voivodeship, almost a quarter, i.e., 23.6% of women filled the prescription for an antibiotic. Most of the prescriptions in general concerned one group of antibiotics, and most often, they were safe beta-lactam antibiotics. However, the data on prescriptions are very often disturbing in the youngest patients and in women not living in cities. Andrade et al. [9] report that as many as 39.8% of all women in the United States received drugs from the group of antimicrobial drugs, either oral or intravenous—almost twice as much as in our study. In this analysis, also in individual trimesters, the percentage of prescriptions was about half lower, although it increased slightly in the second and then third trimesters, and the American study showed similar prescription rates in individual trimesters. The number of antibiotic prescriptions recorded in our study is almost two times lower, probably due to the fact that we only analyzed prescriptions issued by gynecologists and, therefore, related to genital tract and urinary tract infections, and the American study included all orders, therefore also infections other than those mentioned. 

Indications for antibiotic therapy in outpatient pregnancy services include primarily the treatment of ascending infections (bacterial vaginosis, urinary tract infections, sexually transmitted infections) and other infections not related to pregnancy. Schnarr and Smaill report that urinary tract infections are among the most common bacterial infections in pregnant women, as 7.4% of the women in their study were diagnosed as having a urinary tract infection and asymptomatic bacteriuria ranged from 2 to 10% [16]. A meta-analysis by Azami et al. for the population of Iranian pregnant women showed the prevalence of asymptomatic bacteriuria to be 8.7% (95% CI: 7.2–10.4), and urinary tract infections–9.8% (95% CI 7.6–12.5) [21]. 

Bacterial vaginosis, resulting from the imbalance of the bacterial microbiota in pregnant women, as a result of a reduction in the number of Lactobacillus bacteria with a simultaneous increase in the number of anaerobic bacteria and mycoplasmas, can lead to serious complications, including premature births, which Leitich et al. confirmed by a meta-analysis, odds ratio 2.19, 95% confidence level 1.5–3.1 [22,23]. The prevalence of bacterial vaginosis in pregnancy varies depending on the studied population. In a large cohort study of 14,193 women in the Nord-Pas de Calais region in France, the incidence was estimated pursuant to a study based on the Nugent scale at 7.1% (95% CI 6.6–7.5%) [23]. In the Polish population, the prevalence in pregnant women with a normal pregnancy reaches 1.6% and in women with a risk of preterm labor: 14.2% [13,14]. Thus, urinary tract infections and genital tract infections, including bacterial vaginosis, represent an important problem from a public health perspective [13,14,24,25].

In the present data, the antibiotic prescriptions in approximately 23% of pregnant women may indicate that infections of the genital tract and urinary system in the study population were in the middle of the ranges reported by the researchers cited above. However, the association of these data with demographic factors indicate groups of patients requiring special attention and perinatal care. As in the previous study in the same group, but with regard to 30 days from the date of delivery, it can be concluded that the need to use antibiotics is more common in the youngest women, under 18 years of age. The problem of numerous complications of pregnancy in teenagers, such as pregnancy-induced hypertension, anemia, and premature birth, has been confirmed by numerous previous studies [26]. In a study by Oakeshott et al. regarding the relationship between bacterial vaginosis or chlamydial infection and miscarriages before 16 weeks of gestation, the highest prevalence of chlamydial infections in pregnant teenagers was 14.3%, compared to the average of 2.4% for the entire treatment group and 8.5% in women under 25 years of age [27]. Therefore, this group of patients requires special perinatal care, similarly to rural residents, who had the highest percentage of antibiotic orders, taking into account the place of residence. Thus, there are serious gaps in the prevention and control of infections in the youngest pregnant women. Urinary tract infections, which are not only the most common but also the most preventable, can also be a problem. However, this type of intervention requires knowledge of reproductive health and health promotion in general. This calls for an urgent need for periodic educational interviews. The effectiveness of integrated, active, and targeted programs in the prevention of urinary tract infections among pregnant women was confirmed in a randomized clinical trial by Yazdi et al. [25], and Noroozi et al. [28]. If an intrauterine infection is suspected or confirmed, antibiotics are also used [29].

According to the classification developed by the American Food And Drug Administration (FDA), the selection of an antimicrobial drug used during pregnancy should take into account the proposed classification for drugs according to categories: A, B, C, D, and X [30]. Category A includes drugs that have not been shown to be harmful in clinical trials carried out in pregnant women—but none of the antibiotics falls into this category. Category B comprises drugs that have not shown any risk to the fetus in animal studies, but this has not been confirmed in pregnant women. During pregnancy, drugs from group B are recommended, including antibiotics from the penicillin group, cephalosporins, some macrolides (azithromycin, erythromycin), as well as daptomycin (glycolipopeptides), metronidazole, clindamycin, antifungal antibiotics, and terbotericifin. This analysis showed that the vast majority of prescriptions concerned drugs belonging to group B, mainly beta-lactam antibiotics, cephalosporins, and macrolides—about 97% of all prescriptions. However, approximately 1.3% of the prescriptions were for group C fluoroquinolones, i.e., drugs that have shown teratogenic and embryotoxic effects in animal studies and that may only be used when their benefit outweighs the risk of adverse effects in the fetus. Moreover, about 1% of the prescriptions were orders for preparations from the tetracycline group, classified as category D, i.e., drugs for which there is a documented risk to humans. In December 2014, the FDA approved and published a new format and content for the labeling of medicinal products that abolished the original pregnancy risk category system for all prescription drugs, approved since June 2001 [31].

Presenting results have some limitations. Firstly, the database did not include, and the analysis did not encompass, miscarriages. We took into account data on filling the antibiotic prescriptions ordered by gynecologists in the course of the nine months preceding childbirth. Consequently, our results are burdened by a few percent bias. We did not have information about specific indications, but only about prescriptions. However, the value of our analysis is its pioneering nature, and results indicate areas of perinatal care that require more thorough research and intervention.

## 5. Conclusions

Almost a quarter of Polish pregnant women filled a prescription for an antibiotic. The antibiotic prescriptions in approximately 23% of pregnant women may indicate that infections of the genital tract and urinary system in the study population were in the middle of the ranges reported by the most of other studies on this subject. However, an insufficient number of studies on antibiotic therapy in pregnant women make it impossible to assess the situation. Additionally, in Poland, the prevalence of gestational infections is unknown, and their diagnostics is important—especially in relation to microbiological diagnostics and targeted treatment. Most often—94.78% of cases—monotherapy was used, including beta-lactams and beta-lactams with an inhibitor (67.3%). This observation was consistent with the recommendations for antibiotic therapy in pregnancy. However, the present results indicate the need to implement broad health promotion programs, accessible to all women, as well as those living outside large cities, particularly promoting the prevention of urinary tract infections and reproductive health in general. 

## Figures and Tables

**Table 1:** Number of antibiotic prescriptions and DDD (defined daily doses), according to trimesters.

Antibiotic Prescriptions Measure	Total N = 16,059	First Trimester N = 5297	Second Trimester N = 6129	Third Trimester N = 7594
Average number of DDD per woman with prescription (SD)	44.7 (211.8)	37 (124.8)	52.4 (219.5)	26.2 (64.6)
Average number of DDD per delivery (SD)	1056.8 (10,471.9)	291.5 (3625.7)	472.4 (6759.6)	292.9 (2311.1)
Average number of prescriptions (SD)	1.3 (0.70)	1.1 (0.24)	1.1 (0.38)	1.1 (0.38)

DDD—defined daily dose, SD—standard deviation.

**Table 2:** Number of prescriptions and DDD for the whole period of pregnancy according to place of living, hospital type, women’s age, and type of delivery.

Category	Number of Deliveries Total	% of Women with Antibiotic Prescriptions	*p*	DDD/100 Deliveries Average (SD)	*p*
Place of living					
Village	37,268	24.4	*p* < 0.001	1177.9 (11,594.4)	*p* < 0.001
city < 100,000 inhabitants	13,923	23.5		880.4 (7491.9)	
city ≥ 100,000 inhabitants	16,679	21.9		933.9 (9961.5)	
Age					
<18 years	393	32.1	*p* < 0.001	949.0 (2317.7)	*p* < 0.001
18–34 years	57,326	23.8		1039.0 (10,421.5)	
35 years and more	10,198	22.7		1160.7 (10,936.6)	
Type of delivery					
Natural	15,527	23.2	*p* = 0.001	1002.0 (9002.2)	*p* < 0.001
Natural with episiotomy	22,839	23.0		1026.0 (11,008.8)	
Cesarean section	29,551	24.4		1109.3 (10,760.6)	
Type of hospital					
Primary referral hospital	15,875	22.5	*p* < 0.001	923.0 (9053.9)	*p* < 0.001
Secondary referral hospital	46,502	23.7		1009.6 (9710.1)	
Tertiary referral hospital	5540	26.3		1836.1 (17,817.5)	
Delivery					
Single child	52,895	23.4	*p* = 0.004	694.3 (8909.8)	*p* = 0.015
Multiple or preterm birth	1325	26.2		771.0 (3176.5)	
Pregnancy pathology	13,697	24.4		1441.5 (15,363.8)	
Total	67,917	23.6		1056.8 (10,471.9)	

DDD—defined daily dose, *p*—level of significance.

**Table 3:** Number of prescriptions and DDD for first trimester of pregnancy according to place of living, hospital type, women’s age, and type of delivery.

Category	Number of Deliveries Total	% of Women with Antibiotic Prescriptions	*p*	DDD/100 Deliveries Average (SD)	*p*
Place of living					
Village	37,268	8.2	*p* < 0.001	321.9 (3861.6)	*p* < 0.01
city < 100,000 inhabitants	13,923	7.7		249.1 (2640.6)	
city ≥ 100,000 inhabitants	16,679	6.9		259.3 (3792.7)	
Age					
<18 years	393	10.9	*p* = 0.006	289.7 (1082.9)	*p* = 0.006
18–34 years	57,326	7.9		292.9 (3615.3)	
35 years and more	10,198	7.2		283.7 (3745.8)	
Type of delivery					
Natural	15,527	7.3	*p* = 0.001	256.4 (2712.0)	*p* = 0.001
Natural with episiotomy	22,839	7.5		309.2 (4525.8)	
Cesarean section	29,551	8.3		296.3 (3243.2)	
Type of hospital					
Primary referral hospital	15,875	7.5	*p* = 0.017	238.2 (2532.6)	*p* = 0.012
Secondary referral hospital	46,502	7.8		279.3 (3289.6)	
Tertiary referral hospital	5540	8.7		546.6 (7203.0)	
Type of birth					
Single child	52,895	7.7	*p* = 0.030	265.7 (2978.8)	*p* = 0.039
Multiple or preterm birth	1325	8.2		201.6 (999.5)	
Pregnancy pathology	13,697	8.3		399.9 (5550.3)	
Total	67,917	7.8		291.5 (3625.7)	

DDD—defined daily dose, *p*—level of significance.

**Table 4:** Number of prescriptions and DDD for the second trimester according to place of living, hospital type, women’s age, and type of delivery.

Category	Number of Deliveries Total	% of Women with Antibiotic Prescriptions	*p*	DDD/ 100 Deliveries Average (SD)	*p*
Place of living					
Village	37,268	9.2	*p* = 0.111	533.6 (7645.4)	*p* = 0.091
city < 100,000 inhabitants	13,923	9.0		382.4 (5400.6)	
city ≥ 100,000 inhabitants	16,679	8.7		411.0 (5576.8)	
Age					
<18 years	393	12.7	*p* = 0.015	352.5 (1354.1)	*p* = 0.014
18–34 years	57,326	9.1		460.3 (6740.4)	
35 years and more	10,198	8.6		544.7 (6990.2)	
Type of delivery					
Natural	15,527	8.9	*p* = 0.066	449.6 (5536.8)	*p* = 0.070
Natural with episiotomy	22,839	8.8		449.9 (6826.9)	
Cesarean section	29,551	9.3		501.7 (7273.0)	
Type of hospital					
Primary referral hospital	15,875	8.3	*p* < 0.001	413.9 (6277.1)	*p* < 0.001
Secondary referral hospital	46,502	9.0		446.3 (6530.0)	
Tertiary referral hospital	5540	11.2		858.3 (9446.4)	
Type of birth					
Single child	52,895	8.9	*p* = 0.024	418.0 (5778.3)	*p* = 0.026
Multiple or preterm birth	1325	10.1		329.8 (2411.3)	
Pregnancy pathology	13,697	9.5		696.0 (9850.1)	
Total	67,917	9.0		472.4 (6759.9)	

DDD—defined daily dose, *p*—level of significance.

**Table 5:** Number of prescriptions and DDD for the third trimester according to place of living, hospital type, women’s age, and type of delivery.

Category	Number of Deliveries Total	% of Women with Antibiotic Prescriptions	*p*	DDD/ 100 Deliveries Average (SD)	*p*
Place of living					
Village	37,268	11.7	*p* < 0.001	322.3 (2535.0)	*p* < 0.001
city < 100,000 inhabitants	13,923	11.1		249.0 (1570.4)	
city ≥ 100,000 inhabitants	16,679	10.0		263.6 (2307.7)	
Age					
<18 years	393	16.5	*p* = 0.001	307.5 (868.0)	*p* = 0.002
18–34 years	57,326	11.2		285.8 (2219.2)	
35 years and more	10,198	10.8		332.3 (2803.4)	
Type of delivery					
Natural	15,527	11.3	*p* = 0.318	295.9 (2295.1)	*p* = 0.281
Natural with episiotomy	22,839	10.9		266.9 (1995.2)	
Cesarean section	29,551	11.3		311.4 (2536.0)	
Type of hospital					
Primary referral hospital	15,875	10.5	*p* < 0.001	270.9 (2368.6)	*p* < 0.001
Secondary referral hospital	46,502	11.3		283.9 (2076.1)	
Tertiary referral hospital	5,540	12.4		431.3 (3634.4)	
Type of birth					
Single child	52,895	11.1	*p* = 0.158	280.6 (2178.9)	*p* = 0.192
Multiple or preterm birth	1325	12.8		239.6 (967.1)	
Pregnancy pathology	13,697	11.3		345.6 (2838.4)	
Total	67,917	11.2		292.9 (2311.1)	

DDD—defined daily dose, *p*—level of significance.

## Data Availability

The data presented in this study are available on the reasonable request from the corresponding author. The data are not publicly available due to the fact that the database was made available to the authors primarily for the purpose of their analysis and publication of the results in the form of collective studies.
